# An annotated high-content fluorescence microscopy dataset with EGFP-Galectin-3-stained cells and manually labelled outlines

**DOI:** 10.1016/j.dib.2024.111148

**Published:** 2024-11-19

**Authors:** Salma Kazemi Rashed, Malou Arvidsson, Rafsan Ahmed, Sonja Aits

**Affiliations:** Cell Death, Lysosomes and Artificial Intelligence Group, Department of Experimental Medical Science, Faculty of Medicine, Lund University, BMC D10, 22184 Lund, Sweden

**Keywords:** Instance segmentation, Fluorescence microscopy, Biomedical image analysis, High-content screening, Computer vision, Deep learning training and evaluation

## Abstract

Many forms of bioimage analysis involve the detection of objects and their outlines. In the context of microscopy-based high-throughput drug and genomic screening and even in smaller scale microscopy experiments, the objects that most often need to be detected are cells. In order to develop and benchmark algorithms and neural networks that can perform this task, high-quality datasets with annotated cell outlines are needed.

We have created a dataset, named Aitslab_bioimaging2, consisting of 60 fluorescence microscopy images with EGFP-Galectin-3 labelled cells and their hand-labelled outlines. Images were acquired on a Thermo Fischer CX7 high-content imaging system at 20x magnification created as part of an RNA interference screen with a modified U2OS osteosarcoma cell line. Outlines were labelled by three annotators, who had high inter-annotator agreement between them and with a biomedical expert, who labelled some of the objects for comparison and reviewed a subset of the labels, making minor corrections as needed.

The dataset comprises over 2200 annotated cell objects in total, making it sufficient in size to train high-performing neural networks for instance or semantic segmentation. Labels can also easily be converted to boxes for object detection tasks. The dataset is already pre-divided into training, development, and test sets. Matching nuclear staining and outlines are available for part of the dataset from a previous publication (dataset Aitslab_bioimaging1) [1].

Specifications table

**Table:** 

Subject	Computer Vision and Pattern Recognition Bioinformatics
Specific subject area	Analysis of microscopy images*.*
Type of data	Fluorescence microscopy images (C01 and png format, grayscale) and corresponding images with annotated cell objects (png format, RGB) File lists (txt.files) containing the names of the images in training, development and test set Annotation guide (Powerpoint file)
Data collection	60 images were randomly selected from a very large dataset acquired in an RNA interference screen. In the screen, images of modified U2OS cells, which stably expressed EGFP-Galectin-3, were acquired in the green fluorescence channel in a 4 × 4 grid using a high-content imaging system (Thermo Fisher CellInsight CX7 High Content Screening Platform and the associated HCS Studio software) at 20x magnification. For annotation, images were converted from the microscope-generated .C01 format (“original_C01” folder) to 8-bit grayscale .png format (“original_png” folder) and a brighter version of each png image was generated using a previously published script [1] (“normalized_png” folder). Outlines of all cells observed in the images were hand-labelled with the polygon tool of the CVAT annotation software (https://github.com/openvinotoolkit/cvat), following the instructions in the annotation guide. Labelling was performed by three annotators. Selected annotations were double-checked by a senior biomedical and computer vision researcher (Sonja Aits), who also annotated one image for comparison. Annotations were exported as 24-bit rgb png image (Segmentation mask 1.1 format) in which each cell object has a different, randomly assigned color (“annotations” folder). Images were randomly split into training, development and test set at a ratio 36:12:12.
Data source location	**Original fluorescence microscopy images (C01)** Institution: Victorian Centre for Functional Genomics (VCFG), Peter MacCallum Cancer Centre City/Town/Region: Melbourne Country: Australia Latitude and longitude: -37.79845, 144.95645 **Converted and normalized images (png), file lists, annotations and annotation guide** Institution: Biomedical Centre (BMC), Lund University City/Town/Region: Lund Country: Sweden Latitude and longitude: 55.71264, 13.20156*.*
Data accessibility	Repository name: zenodo Data identification number: (DOI: 10.5281/zenodo.12193890) Direct URL to data: (https://doi.org/10.5281/zenodo.12193890) The C01 to png conversion script and instructions for its use is available from a prior publication [1] and on GitHub (https://github.com/Aitslab/bioimaging/tree/main/C01_conversion). The CVAT annotation software (not created by us) with which the images were annotated is available from https://github.com/cvat-ai/cvat.
Related research article	*None*

## 1 Value of the Data

• The dataset is of value for anyone who wants to develop or evaluate algorithms or neural networks for instance or semantic segmentation, and especially for researchers working on this for cell detection in microscopy images. The annotations can also easily be converted to bounding boxes when object detection is sufficient.
• The number of objects and images in the dataset is sufficient for it to serve as both training and benchmarking dataset.
• For most images in the dataset, corresponding nuclear staining images and outlines are available from a previous publication [1]. The two datasets can be combined to develop algorithms and neural networks that can detect both object types at the same time. Few similar datasets with outlines for both object types are currently available.
• To improve generalization, the dataset can also be used in combination with other annotated datasets. An example of a similar cell outline dataset is the BBBC020 dataset (available from https://bbbc.broadinstitute.org/BBBC020; accessed on June 20, 2024)
• The involvement of a senior biomedical researcher with computer vision expertise in the production of the dataset ensures high quality.

## 2 Background

Analysis of fluorescence microscopy often involves the quantification of cellular phenotypes (e.g. cell size, cell shape, localization or level of proteins of interest in the cells). For this, it is essential to accurately detect the cells as regions of interest in the image. Automation of this cell detection step is desirable to increase throughput and reproducibility, and this can be achieved with deep learning models or other algorithms that perform instance or semantic segmentation. The development and evaluation of such models or algorithms requires datasets with hand-labelled annotations of the cell outlines as “ground truth”. Only a few such annotated datasets have been produced due to the required effort. Here, we created a new dataset with annotated cell outlines using images of U2OS cells expressing EGFP-Galectin-3, which had been acquired for a high-content microscopy screen. EGFP-Galectin-3 was chosen to increase the difficulty of the cell detection as it only labels the inside of the cells without highlighting the plasma membrane. The dataset was created with the help of a senior researcher with combined expertise in biomedical research and computer vision to ensure high quality annotations.

## 3 Data Description

### 3.1 Images

The microscopy image dataset consists of 60 fluorescence microscopy images of siRNA-transfected EGFP-Galectin-3 expressing U2OS cells in three formats: as original files in .C01 format (folder “original_C01”), converted images in .png format (folder “original_png”) and normalized images with increased brightness in .png format (folder “normalized_png”).

The file names contain a 12-digit plate ID (e.g. 170702090001), the well position (e.g. A12) and image position (e.g. f02), which refers to the position inside the well at which the image was taken, and the fluorescence channel used for imaging (d1).

For each microscopy image there is one or several corresponding images with the annotations of the cell objects (in .png format), with each object shown in a randomly assigned color (folder “annotations”). Annotation files have name of the original file, followed by the initials of the annotator as suffix (“_SR” for Salma Kazemi Rashed, “_MA” for Malou Arvidsson, “_RA” for Rafsan Ahmed and “_SA” for the biomedical expert Sonja Aits). Several images were annotated by more than one annotator for comparison.

**Fig. 1:**
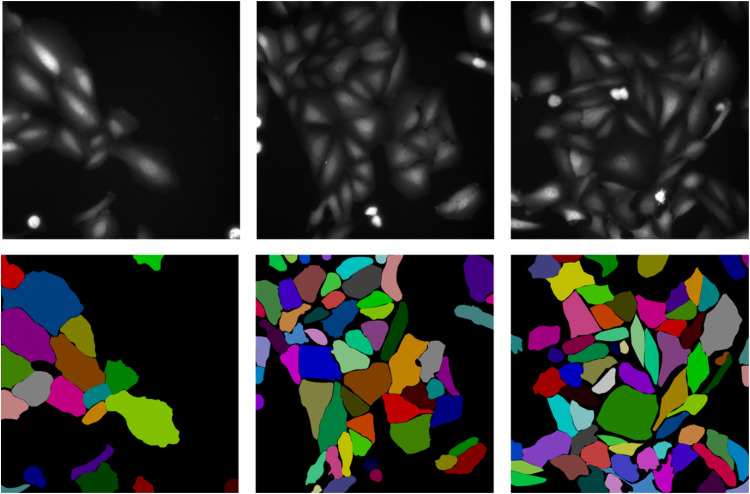
Example microscopy images and corresponding cell annotations. Three representative images of EGFP-Galectin-3 expressing U2OS cells are shown on top. Annotations for each image with randomly assigned colors for each object are shown in the bottom.

The image dataset was grouped into zip folders for the training, development and test subset at a ratio of 36:12:12. Multiple annotations for the same image are kept in the same subset to avoid data leakage. (Fig. 1)

*Training set (training_cells.zip)*

**Table:** 

Original microscopy≥ images (folder “original_C01”)	Converted images (folder “original_png”)	Normalized images (folder “normalized_png”)	Annotation images (folder “annotations”)
MFGTMPcx7_170702000001_ G07f02d1.C01	MFGTMPcx7_170702000001_ G07f02d1.png	MFGTMPcx7_170702000001_ G07f02d1.png	MFGTMPcx7_170702000001_ G07f02d1_MA.png
MFGTMPcx7_170702090001_ A02f07d1.C01	MFGTMPcx7_170702090001_ A02f07d1.png	MFGTMPcx7_170702090001_ A02f07d1.png	MFGTMPcx7_170702090001_ A02f07d1_MA.png
MFGTMPcx7_170702090001_ A08f12d1.C01	MFGTMPcx7_170702090001_ A08f12d1.png	MFGTMPcx7_170702090001_ A08f12d1.png	MFGTMPcx7_170702090001_ A08f12d1_MA.png
MFGTMPcx7_170702090001_ A10f11d1.C01	MFGTMPcx7_170702090001_ A10f11d1.png	MFGTMPcx7_170702090001_ A10f11d1.png	MFGTMPcx7_170702090001_ A10f11d1_MA.png
MFGTMPcx7_170702090001_ A12f00d1.C01	MFGTMPcx7_170702090001_ A12f00d1.png	MFGTMPcx7_170702090001_ A12f00d1.png	MFGTMPcx7_170702090001_ A12f00d1_MA.png
MFGTMPcx7_170702090001_ B08f09d1.C01	MFGTMPcx7_170702090001_ B08f09d1.png	MFGTMPcx7_170702090001_ B08f09d1.png	MFGTMPcx7_170702090001_ B08f09d1_MA.png
MFGTMPcx7_170702090001_ C09f01d1.C01	MFGTMPcx7_170702090001_ C09f01d1.png	MFGTMPcx7_170702090001_ C09f01d1.png	MFGTMPcx7_170702090001_ C09f01d1_MA.png
MFGTMPcx7_170702090001_ C16f04d1.C01	MFGTMPcx7_170702090001_ C16f04d1.png	MFGTMPcx7_170702090001_ C16f04d1.png	MFGTMPcx7_170702090001_ C16f04d1_MA.png
MFGTMPcx7_170702090001_ F20f14d1.C01	MFGTMPcx7_170702090001_ F20f14d1.png	MFGTMPcx7_170702090001_ F20f14d1.png	MFGTMPcx7_170702090001_ F20f14d1_MA.png
MFGTMPcx7_170702090001_ H03f10d1.C01	MFGTMPcx7_170702090001_ H03f10d1.png	MFGTMPcx7_170702090001_ H03f10d1.png	MFGTMPcx7_170702090001_ H03f10d1_MA.png
MFGTMPcx7_170702090001_ K22f04d1.C01	MFGTMPcx7_170702090001_ K22f04d1.png	MFGTMPcx7_170702090001_ K22f04d1.png	MFGTMPcx7_170702090001_ K22f04d1_SK.png
MFGTMPcx7_170702090001_ L21f03d1.C01	MFGTMPcx7_170702090001_ L21f03d1.png	MFGTMPcx7_170702090001_ L21f03d1.png	MFGTMPcx7_170702090001_ L21f03d1_SK.png
MFGTMPcx7_170702090001_ N06f14d1.C01	MFGTMPcx7_170702090001_ N06f14d1.png	MFGTMPcx7_170702090001_ N06f14d1.png	MFGTMPcx7_170702090001_ N06f14d1_SK.png
MFGTMPcx7_170702090001_ O02f15d1.C01	MFGTMPcx7_170702090001_ O02f15d1.png	MFGTMPcx7_170702090001_ O02f15d1.png	MFGTMPcx7_170702090001_ O02f15d1_SK.png
MFGTMPcx7_170702090001_ P08f09d1.C01	MFGTMPcx7_170702090001_ P08f09d1.png	MFGTMPcx7_170702090001_ P08f09d1.png	MFGTMPcx7_170702090001_ P08f09d1_SK.png
MFGTMPcx7_170731090001_ B05f10d1.C01	MFGTMPcx7_170731090001_ B05f10d1.png	MFGTMPcx7_170731090001_ B05f10d1.png	MFGTMPcx7_170731090001_ B05f10d1_SK.png
MFGTMPcx7_170731090001_ B14f13d1.C01	MFGTMPcx7_170731090001_ B14f13d1.png	MFGTMPcx7_170731090001_ B14f13d1.png	MFGTMPcx7_170731090001_ B14f13d1_SK.png
MFGTMPcx7_170731090001_ G15f00d1.C01	MFGTMPcx7_170731090001_ G15f00d1.png	MFGTMPcx7_170731090001_ G15f00d1.png	MFGTMPcx7_170731090001_ G15f00d1_SK.png
MFGTMPcx7_170731090001_ G15f03d1.C01	MFGTMPcx7_170731090001_ G15f03d1.png	MFGTMPcx7_170731090001_ G15f03d1.png	MFGTMPcx7_170731090001_ G15f03d1_MA.png MFGTMPcx7_170731090001_ G15f03d1_SK.png
MFGTMPcx7_170731090001_ I12f02d1.C01	MFGTMPcx7_170731090001_ I12f02d1.png	MFGTMPcx7_170731090001_ I12f02d1.png	MFGTMPcx7_170731090001_ I12f02d1_SK.png
MFGTMPcx7_170731090001_ I12f07d1.C01	MFGTMPcx7_170731090001_ I12f07d1.png	MFGTMPcx7_170731090001_ I12f07d1.png	MFGTMPcx7_170731090001_ I12f07d1_MA.png MFGTMPcx7_170731090001_ I12f07d1_SK.png
MFGTMPcx7_170731090001_ K05f07d1.C01	MFGTMPcx7_170731090001_ K05f07d1.png	MFGTMPcx7_170731090001_ K05f07d1.png	MFGTMPcx7_170731090001_ K05f07d1_MA.png MFGTMPcx7_170731090001_ K05f07d1_SK.png
MFGTMPcx7_170731090001_ K24f09d1.C01	MFGTMPcx7_170731090001_ K24f09d1.png	MFGTMPcx7_170731090001_ K24f09d1.png	MFGTMPcx7_170731090001_ K24f09d1_SK.png
MFGTMPcx7_170731090001_ K24f10d1.C01	MFGTMPcx7_170731090001_ K24f10d1.png	MFGTMPcx7_170731090001_ K24f10d1.png	MFGTMPcx7_170731090001_ K24f10d1_SK.png
MFGTMPcx7_170801050001_ A01f03d1.C01	MFGTMPcx7_170801050001_ A01f03d1.png	MFGTMPcx7_170801050001_ A01f03d1.png	MFGTMPcx7_170801050001_ A01f03d1_SK.png
MFGTMPcx7_170802000001_ I12f01d1.C01	MFGTMPcx7_170802000001_ I12f01d1.png	MFGTMPcx7_170802000001_ I12f01d1.png	MFGTMPcx7_170802000001_ I12f01d1_SK.png
MFGTMPcx7_170803210001_ P17f28d1.C01	MFGTMPcx7_170803210001_ P17f28d1.png	MFGTMPcx7_170803210001_ P17f28d1.png	MFGTMPcx7_170803210001_ P17f28d1_SK.png
MFGTMPcx7_170607100001_ K23f08d1.C01	MFGTMPcx7_170607100001_ K23f08d1.png	MFGTMPcx7_170607100001_ K23f08d1.png	MFGTMPcx7_170607100001_ K23f08d1_RA.png
MFGTMPcx7_170623010001_ C23f05d1.C01	MFGTMPcx7_170623010001_ C23f05d1.png	MFGTMPcx7_170623010001_ C23f05d1.png	MFGTMPcx7_170623010001_ C23f05d1_RA.png
MFGTMPcx7_170607150001_ C23f05d1.C01	MFGTMPcx7_170607150001_ C23f05d1.png	MFGTMPcx7_170607150001_ C23f05d1.png	MFGTMPcx7_170607150001_ C23f05d1_RA.png
MFGTMPcx7_170718220001_ C02f06d1.C01	MFGTMPcx7_170718220001_ C02f06d1.png	MFGTMPcx7_170718220001_ C02f06d1.png	MFGTMPcx7_170718220001_ C02f06d1_RA.png
MFGTMPcx7_170729090001_ C02f01d1.C01	MFGTMPcx7_170729090001_ C02f01d1.png	MFGTMPcx7_170729090001_ C02f01d1.png	MFGTMPcx7_170729090001_ C02f01d1_RA.png
MFGTMPcx7_170720170001_ P02f02d1.C01	MFGTMPcx7_170720170001_ P02f02d1.png	MFGTMPcx7_170720170001_ P02f02d1.png	MFGTMPcx7_170720170001_ P02f02d1_RA.png

*Development set (development_cells.zip)*

**Table:** 

Original microscopy images (folder “original_C01”)	Converted images (folder “original_png”)	Normalized images (folder “normalized_png”)	Annotation images (folder “annotations”)
MFGTMPcx7_170702000001_ B23f07d1.C01	MFGTMPcx7_170702000001_ B23f07d1.png	MFGTMPcx7_170702000001_ B23f07d1.png	MFGTMPcx7_170702000001_ B23f07d1_MA.png
MFGTMPcx7_170702000001_ F11f10d1.C01	MFGTMPcx7_170702000001_ F11f10d1.png	MFGTMPcx7_170702000001_ F11f10d1.png	MFGTMPcx7_170702000001_ F11f10d1_MA.png
MFGTMPcx7_170702090001_ A08f09d1.C01	MFGTMPcx7_170702090001_ A08f09d1.png	MFGTMPcx7_170702090001_ A08f09d1.png	MFGTMPcx7_170702090001_ A08f09d1_MA.png
MFGTMPcx7_170702090001_ A20f02d1.C01	MFGTMPcx7_170702090001_ A20f02d1.png	MFGTMPcx7_170702090001_ A20f02d1.png	MFGTMPcx7_170702090001_ A20f02d1_MA.png
MFGTMPcx7_170702090001_ G03f02d1.C01	MFGTMPcx7_170702090001_ G03f02d1.png	MFGTMPcx7_170702090001_ G03f02d1.png	MFGTMPcx7_170702090001_ G03f02d1_MA.png
MFGTMPcx7_170702090001_ K22f14d1.C01	MFGTMPcx7_170702090001_ K22f14d1.png	MFGTMPcx7_170702090001_ K22f14d1.png	MFGTMPcx7_170702090001_ K22f14d1_SK.png
MFGTMPcx7_170702090001_ P01f02d1.C01	MFGTMPcx7_170702090001_ P01f02d1.png	MFGTMPcx7_170702090001_ P01f02d1.png	MFGTMPcx7_170702090001_ P01f02d1_SK.png
MFGTMPcx7_170731090001_ A01f04d1.C01	MFGTMPcx7_170731090001_ A01f04d1.png	MFGTMPcx7_170731090001_ A01f04d1.png	MFGTMPcx7_170731090001_ A01f04d1_SK.png
MFGTMPcx7_170731090001_ B05f12d1.C01	MFGTMPcx7_170731090001_ B05f12d1.png	MFGTMPcx7_170731090001_ B05f12d1.png	MFGTMPcx7_170731090001_ B05f12d1_SK.png
MFGTMPcx7_170802000001_ I10f05d1.C01	MFGTMPcx7_170802000001_ I10f05d1.png	MFGTMPcx7_170802000001_ I10f05d1.png	MFGTMPcx7_170802000001_ I10f05d1_SK.png
MFGTMPcx7_170624160001_ C23f08d1.C01	MFGTMPcx7_170624160001_ C23f08d1.png	MFGTMPcx7_170624160001_ C23f08d1.png	MFGTMPcx7_170624160001_ C23f08d1_RA.png
MFGTMPcx7_170802050001_ H02f01d1.C01	MFGTMPcx7_170802050001_ H02f01d1.png	MFGTMPcx7_170802050001_ H02f01d1.png	MFGTMPcx7_170802050001_ H02f01d1_RA.png

*Test set (test_cells.zip)*

**Table:** 

Original microscopy images (folder “original_C01”)	Converted images (folder “original_png”)	Normalized images (folder “normalized_png”)	Annotation images (folder “annotations”)
MFGTMPcx7_170531000001_ F23f00d1.C01	MFGTMPcx7_170531000001_ F23f00d1.png	MFGTMPcx7_170531000001_ F23f00d1.png	MFGTMPcx7_170702000001_ G14f03d1_MA.png
MFGTMPcx7_170607050001_ I02f15d1.C01	MFGTMPcx7_170607050001_ I02f15d1.png	MFGTMPcx7_170607050001_ I02f15d1.png	MFGTMPcx7_170702090001_ B22f15d1_MA.png
MFGTMPcx7_170702000001_ G14f03d1.C01	MFGTMPcx7_170702000001_ G14f03d1.png	MFGTMPcx7_170702000001_ G14f03d1.png	MFGTMPcx7_170702090001_ C08f14d1_MA.png
MFGTMPcx7_170702090001_ B22f15d1.C01	MFGTMPcx7_170702090001_ B22f15d1.png	MFGTMPcx7_170702090001_ B22f15d1.png	MFGTMPcx7_170702090001_ H04f01d1_SK.png
MFGTMPcx7_170702090001_ C08f14d1.C01	MFGTMPcx7_170702090001_ C08f14d1.png	MFGTMPcx7_170702090001_ C08f14d1.png	MFGTMPcx7_170702090001_ P07f14d1_SK.png
MFGTMPcx7_170702090001_ H04f01d1.C01	MFGTMPcx7_170702090001_ H04f01d1.png	MFGTMPcx7_170702090001_ H04f01d1.png	MFGTMPcx7_170731090001_ B14f09d1_SK.png
MFGTMPcx7_170702090001_ P07f14d1.C01	MFGTMPcx7_170702090001_ P07f14d1.png	MFGTMPcx7_170702090001_ P07f14d1.png	MFGTMPcx7_170731090001_ D11f13d1_SK.png
MFGTMPcx7_170731090001_ B14f09d1.C01	MFGTMPcx7_170731090001_ B14f09d1.png	MFGTMPcx7_170731090001_ B14f09d1.png	MFGTMPcx7_170731090001_ I12f05d1_MA.png MFGTMPcx7_170731090001_ I12f05d1_SK.png
MFGTMPcx7_170731090001_ D11f13d1.C01	MFGTMPcx7_170731090001_ D11f13d1.png	MFGTMPcx7_170731090001_ D11f13d1.png	MFGTMPcx7_170801050001_ G02f01d1_SK.png
MFGTMPcx7_170731090001_ I12f05d1.C01	MFGTMPcx7_170731090001_ I12f05d1.png	MFGTMPcx7_170731090001_ I12f05d1.png	MFGTMPcx7_170803210001_ J12f29d1_SK.png
MFGTMPcx7_170801050001_ G02f01d1.C01	MFGTMPcx7_170801050001_ G02f01d1.png	MFGTMPcx7_170801050001_ G02f01d1.png	MFGTMPcx7_170531000001_ F23f00d1_RA.png
MFGTMPcx7_170803210001_ J12f29d1.C01	MFGTMPcx7_170803210001_ J12f29d1.png	MFGTMPcx7_170803210001_ J12f29d1.png	MFGTMPcx7_170607050001_ I02f15d1_RA.png MFGTMPcx7_170607050001_ I02f15d1_SA.png MFGTMPcx7_170607050001_ I02f15d1_SK.png

### 3.2 File lists

3 text files listing the names of the images in each subset: train_cells.txt development_cells.txt test_cells.txt

### 3.3 Annotation guide

The annotation_guide.pptx file contains the annotation rules and an annotation example.

## 4 Experimental Design, Materials and Methods

The modified U2OS cells, which stably expressed EGFP-Galectin-3, were plated in black 384-well plates with clear bottom (Greiner) and transfected with siRNAs (Dharmacon siGENOME library) for a large genomic screen. The next day, cells were washed and received fresh medium. 72 h after transfection, cells were fixed and stained with Hoechst 33342 in parallel. Sealed plates were stored at 4° prior to image acquisition with a CX7 high-content imaging system (Thermo Fisher) and HCS studio software at 20x magnification in the green fluorescence channel. From each well, 16 images were taken in a non-overlapping grid.

**Fig. 2:**
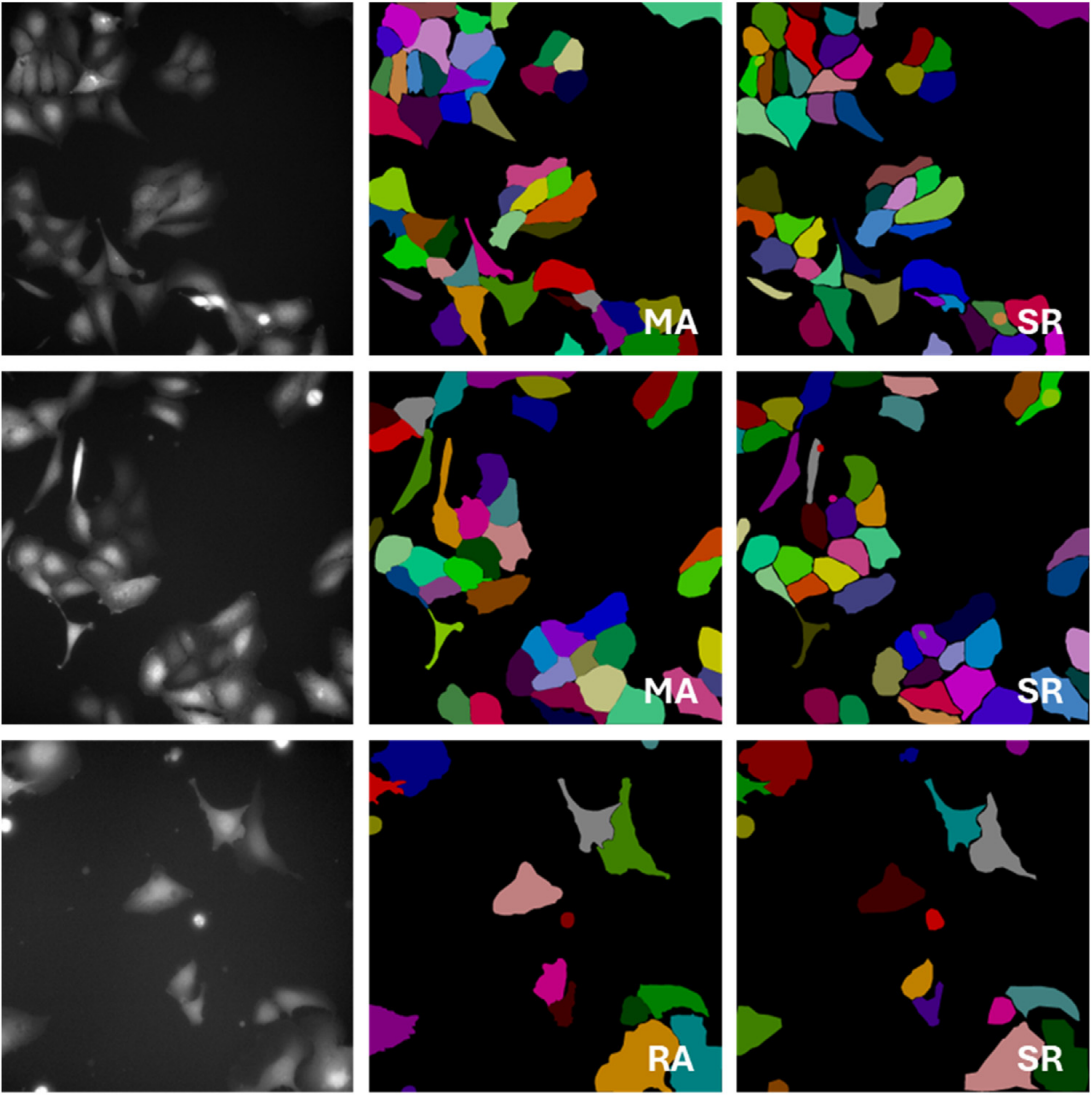
Examples of inter-annotator differences. Fluorescent microscopy images of EGFP-Galectin-3 expressing U2OS cells are shown on the left and corresponding cell outlines labelled by two annotators (indicated by their initials) are shown in the middle and on the right. Only the shape of the objects should be compared as object colors were assigned randomly.

60 images from several 384-well plates in the microscope-generated .C01 format were selected randomly. Prior to annotation, .C01 images were transformed to 8-bit png images and normalized to obtain brighter images using the C01_to_png.py script as described in [1] and the normalize.py script. Outlines of cells visible in the png images were annotated with the CVAT annotation software (https://github.com/cvat-ai/cvat) as a single class using the polygon tool. Annotations were made following the annotation guide by three researchers (SR, RA and MA), who had been trained by a senior biomedical and computer vision researcher (SA). The senior biomedical expert also annotated one image for comparison and reviewed a selection of the annotations, making small corrections. From the images annotated by multiple annotators (Fig. 2), inter-annotator agreement was calculated using the Jaccard index, which exceeded 0.75. Annotations were exported from CVAT as 24-bit rgb png image (Segmentation mask 1.1 format) showing each cell object in a randomly assigned color.

## Limitations

All images are from the same experiment and acquired on the same microscope. When generalization is important the dataset should thus be combined with other datasets.

We chose a cell staining which does not highlight the plasma membrane because this is a more challenging task and because many microscopy experiments do not include a plasma membrane staining. Consequently, the cell border was not always clearly visible and annotations of the outline differed slightly from annotator to annotator.

## Ethics Statement

The authors have read and follow the ethical requirements for publication in Data in Brief. The current work does not involve human subjects, animal experiments, or any data collected from social media platforms. A commercially available human cancer cell line was used for this study, requiring no ethical permit.

## CRediT Author Statement

**Sonja Aits:** Conceptualization, Methodology, Validation, Investigation, Resources, Data curation, Writing, Supervision (lead), Project administration, Funding acquisition, Visualization; **Salma Kazemi Rashed**: Software, Investigation Supervision (supporting), Data curation; **Malou Arvidsson**: Investigation; **Rafsan Ahmed:** Investigation.

## Data Availability

zenodoAn annotated high-content fluorescence microscopy dataset with Hoechst 33342-stained nuclei and manually labelled outlines (Original data)

## References

[1] Arvidsson M., Rashed S.K., Aits S. (2023). An annotated high-content fluorescence microscopy dataset with Hoechst 33342-stained nuclei and manually labelled outlines. Data Brief.

